# Supplementary material for: Chlorambucil targets BRCA1/2‐deficient tumours and counteracts PARP inhibitor resistance
Source: EMBO Mol Med. 2019 May 24;11(7):e9982. doi: 10.15252/emmm.201809982 (PMC6609913; doi:10.15252/emmm.201809982)
Supplement: Supplementary file 1 — Appendix [file EMMM-11-e9982-s001.pdf]

Chlorambucil targets BRCA1/2-deficient tumours and counteracts PARP inhibitor resistance

Eliana M. C. Tacconi, Sophie Badie, Giuliana De Gregoriis, Timo Reisländer, Xianning Lai, Manuela Porru, Cecilia Folio, John Moore, Arnaud Kopp, Júlia Baguña Torres, Deborah Sneddon, Marcus Green, Simon Dedic, Jonathan W. Lee, Ankita Sati Batra, Oscar M. Rueda, Alejandra Bruna, Carlo Leonetti, Carlos Caldas, Bart Cornelissen, Laurent Brino, Anderson Ryan, Annamaria Biroccio and Madalena Tarsounas.

Appendix Contents:

Appendix Figure S1 - Analysis of Prestwick chemical library screens performed in BRCA2-deficient and -proficient hamster cell lines. **Page 2**

Appendix Figure S2 - Melphalan sensitivity of BRCA1/2-proficient and -deficient human cell lines. **Page 3**

Appendix Figure S3 - Chlorambucil/cisplatin induce checkpoint and apoptotic responses in human cells. **Page 4**

Appendix Figure S4 - Effect of chlorambucil and cisplatin on the viability of human H1299 cells upon depletion of FANCD2 and/or XPF. **Page 5**

Appendix Figure S5 - Dose dependent effect of chlorambucil on the growth of BRCA2-deficient xenografts. **Page 6**

Appendix Figure S6 - Cisplatin and chlorambucil sensitivity assessed in BRCA2-proficient and -deficient human HCT116 cells and xenografts. **Page 7**

Appendix Table S1 - Figures Exact P-values. **Page 8**

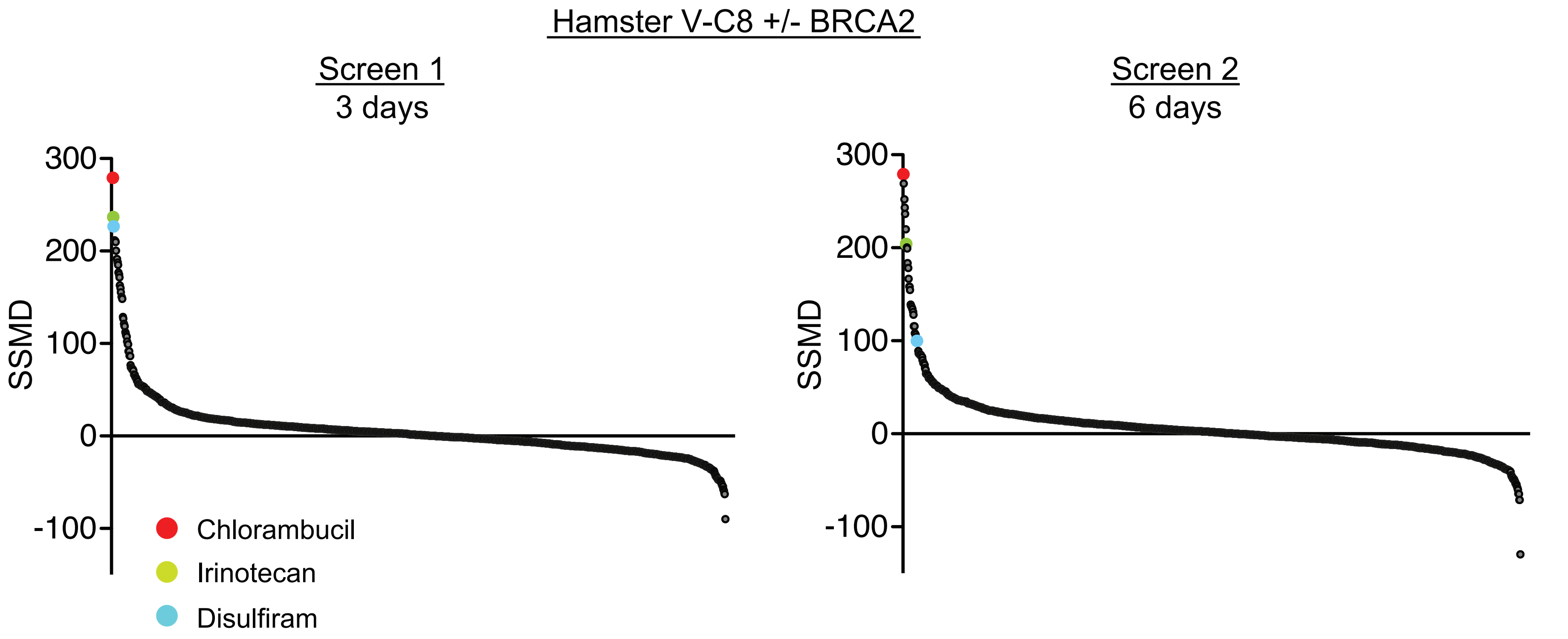

Figure S1. Analysis of Prestwick chemical library screens performed in BRCA2-deficient and -proficient hamster cell lines. Two independent screens were performed, each in triplicate. BRCA2-deficient and -reconstituted hamster cells were incubated with the library of drugs at 5 µM concentration for 3 (Screen 1) or 6 (Screen 2) days. Cell viability was determined using resazurin-based assays. Following plate normalisation, hits were ranked using strictly standardised mean deviation (SSMD); see also Table S1.

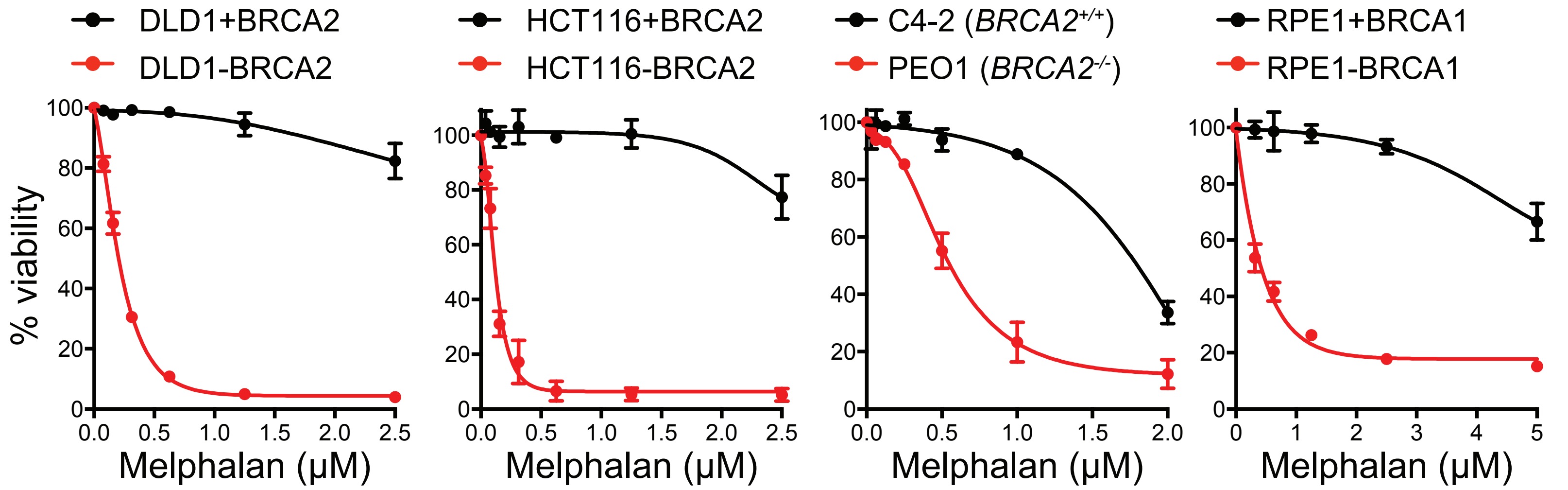

Figure S2. Melphalan sensitivity of BRCA1/2-proficient and -deficient human cell lines. Dose-dependent viability assays of cells treated with melphalan at the indicated concentrations for six days. Graphs represent averages of three independent experiments, each performed in triplicate. Error bars represent SEM.

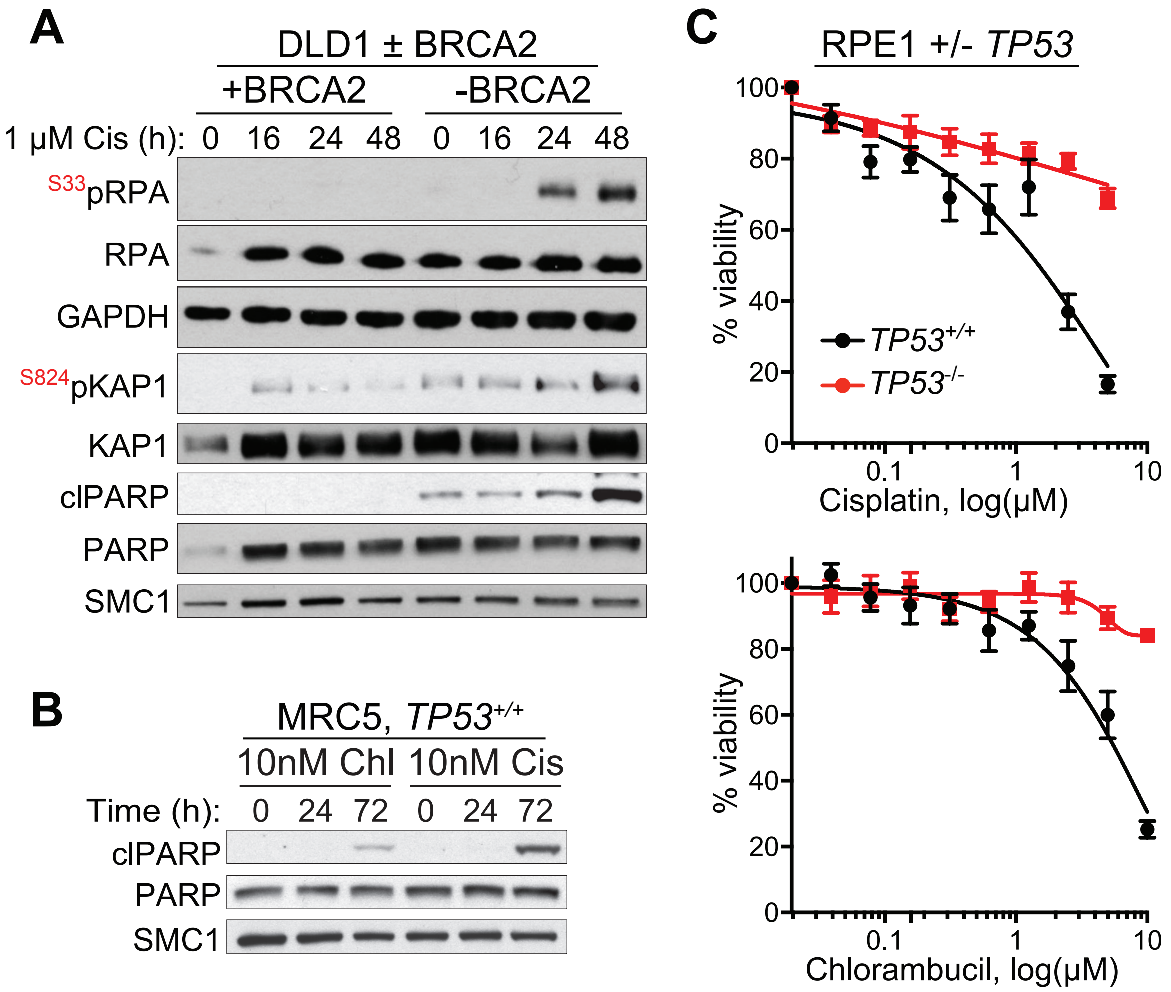

Figure S3. Chlorambucil/cisplatin induce checkpoint and apoptotic responses in human cells. A, BRCA2-proficient (+BRCA2) or -deficient (-BRCA2) human DLD1 cells were incubated with 1µM cisplatin (Cis). Whole cell extracts prepared at the indicated time points during treatment were immunoblotted as shown. GAPDH and SMC1 were used as a loading controls. **B,** Human MRC5 cells were incubated with 10 nM chlorambucil or 10 nM cisplatin. Whole cell extracts prepared at the indicated time points during treatment were immunoblotted as shown. SMC1 was used as a loading control. Cis, cisplatin; Chl, chlorambucil. **C.** Dose-dependent viability assays of p53-proficient (*TP53*$^{+/+}$) or -deficient (*TP53*$^{-/-}$) human RPE1 cells treated with drugs at the indicated concentrations of drug for six days. Graphs represent averge values obtained from three independent experiments, each performed in triplicate. Error bars represent SEM.

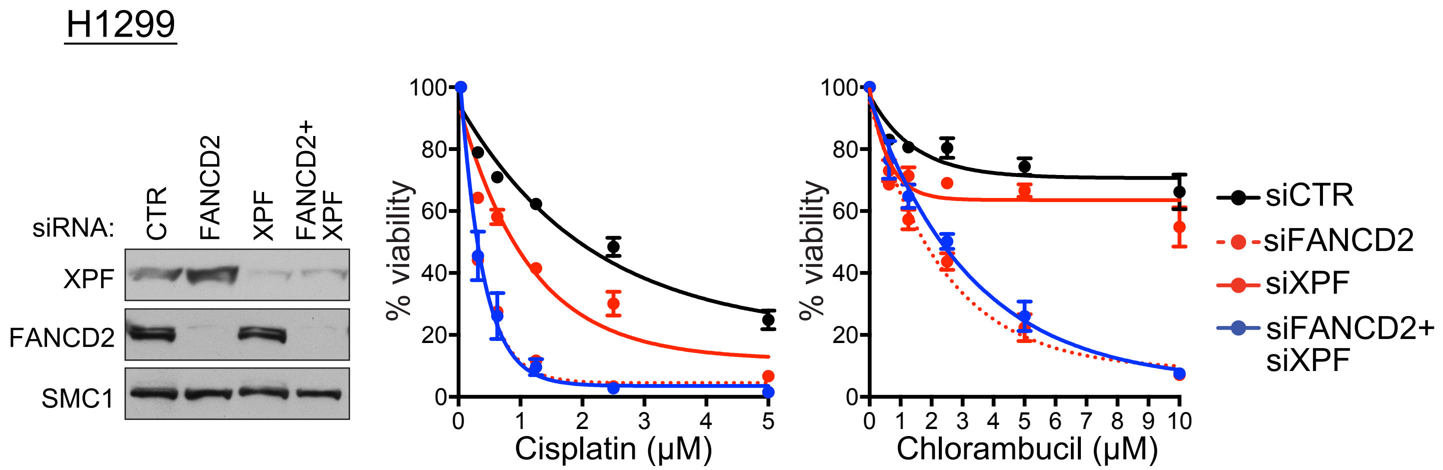

Figure S4. Effect of chlorambucil and cisplatin on the viability of human H1299 cells upon depletion of FANCD2 and/or XPF. Human H1299 cells were treated with control (CTR) or indicated siRNAs two days before drugs were added to the media for dose-dependent viability assays. Cell extracts prepared at the time of drug addition were immunoblotted as indicated. SMC1 was used as a loading control. Graphs represent average values obtained from three independent experiments, each performed intriplicate. Error bars represent SEM.

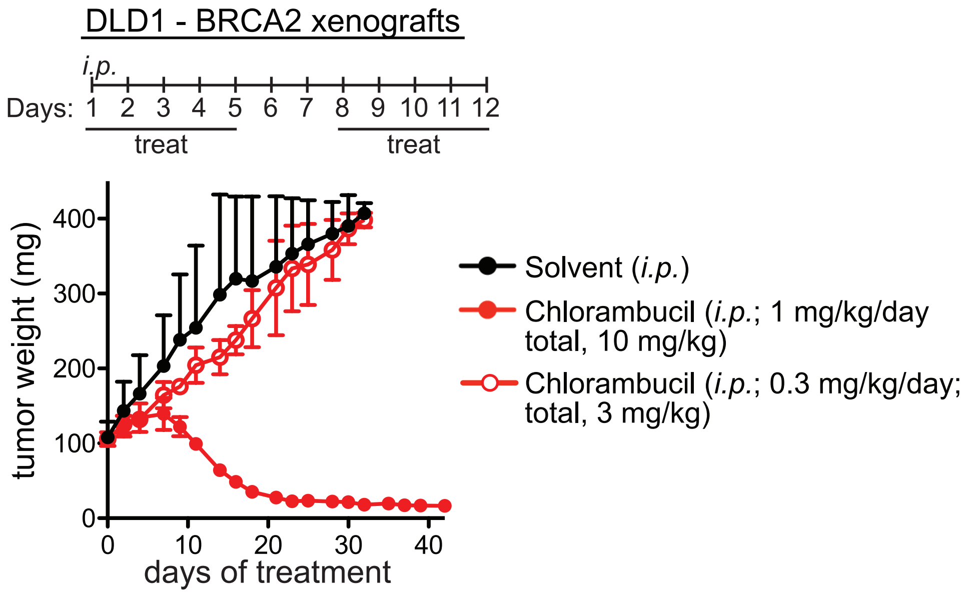

Figure S5. Dose-dependent effect of chlorambucil on the growth of BRCA2-deficient xenografts. Mice were injected intramuscularly with 5 x 10^6 human BRCA2-deficient DLD1 cells. Tumour-bearing mice were treated with 0.3 or 1 mg/kg daily chlorambucil administered intraperitoneally (*i.p.*). Tumour weight was evaluated based on volume, assessed on the indicated days after initiation of the treatment. Each experimental group included four mice. Error bars represent SEM.

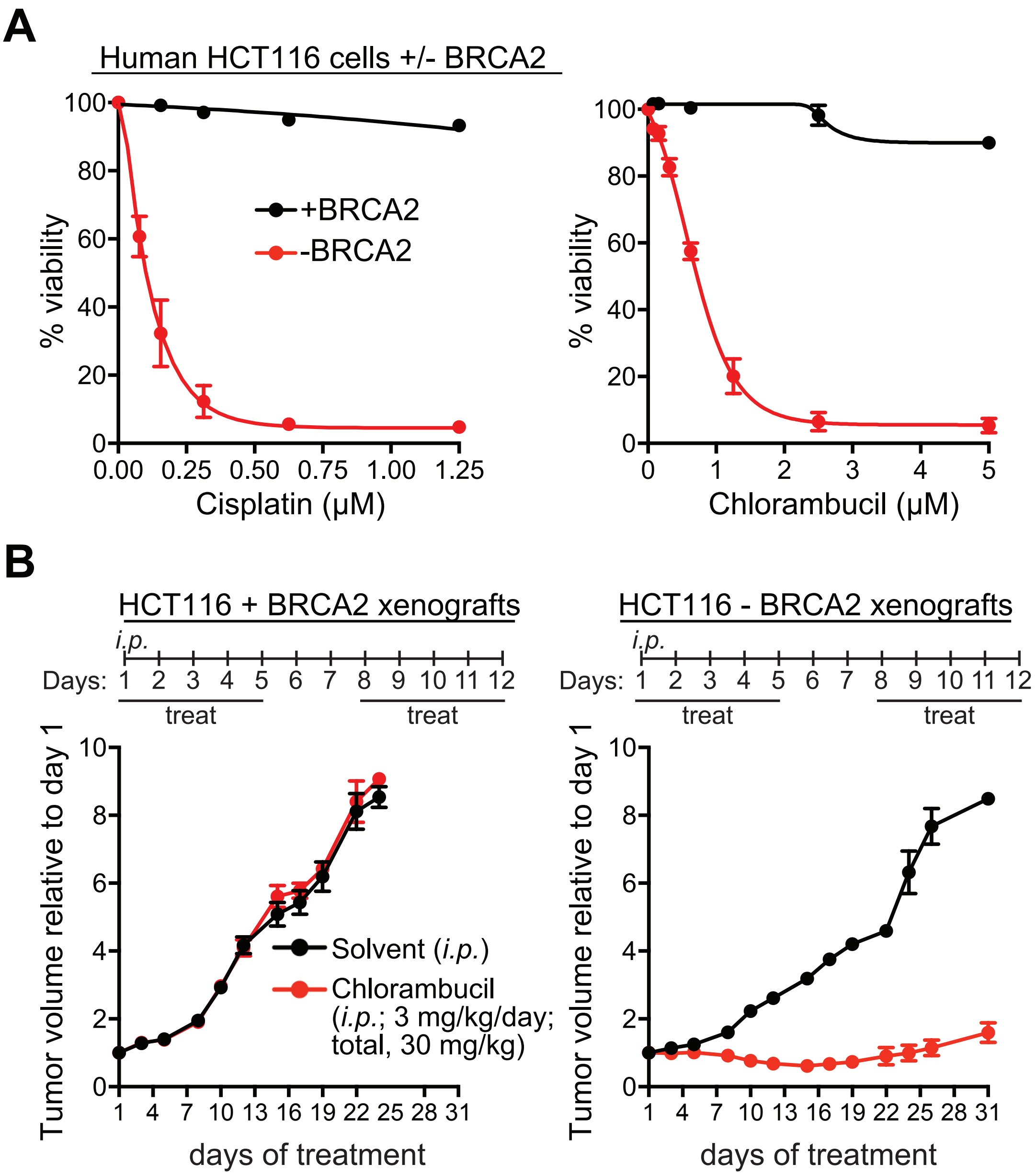

Figure S6. Cisplatin and chlorambucil sensitivity assessed in BRCA2-proficient and -deficient human HCT116 cells and xenografts. A, Dose-dependent viability assays were performed using BRCA2-proficient (+BRCA2) or -deficient (-BRCA2) human HCT116 cells treated with drugs at the indicated concentrations for six days. Graphs represent average values obtained from three independent experiments, each performed in triplicate. Error bars represent SEM. **B,** Mice were injected intramuscularly with 5 x 10^6 human HCT116 cells, BRCA2-proficient or -deficient. Tumour-bearing mice were treated with 3 mg/kg daily chlorambucil administered intraperitoneally. Tumour weight was assessed on the indicated days after initiation of the treatment. Each experimental group included five mice. Error bars represent SEM.

Appendix Table S1

Exact *P*-values for each figure
(If *P*-value was <0.0001 the exact value is not given by the analysis software)

Figure	Compared pairs	*P*-value	Indication
4C	+BRCA2 Cis vs -BRCA2 Cis	0.0025	**
4C	-BRCA2 Cis vs -BRCA2 Chl	0.0045	**
5D	SOLV vs CIS	0.0009	***
6A	Blood Chlorambucil vs Blood Cisplatin	0.0452	*
6A	Lung Chlorambucil vs Lung Cisplatin	0.0285	*
6B	Heart Solvent vs Heart Chlorambucil	0.0211	*
