# Supplementary figures and images for: Chlorambucil targets BRCA1/2‐deficient tumours and counteracts PARP inhibitor resistance
Source: EMBO Mol Med. 2019 May 24;11(7):e9982. doi: 10.15252/emmm.201809982 (PMC6609913; doi:10.15252/emmm.201809982)

A

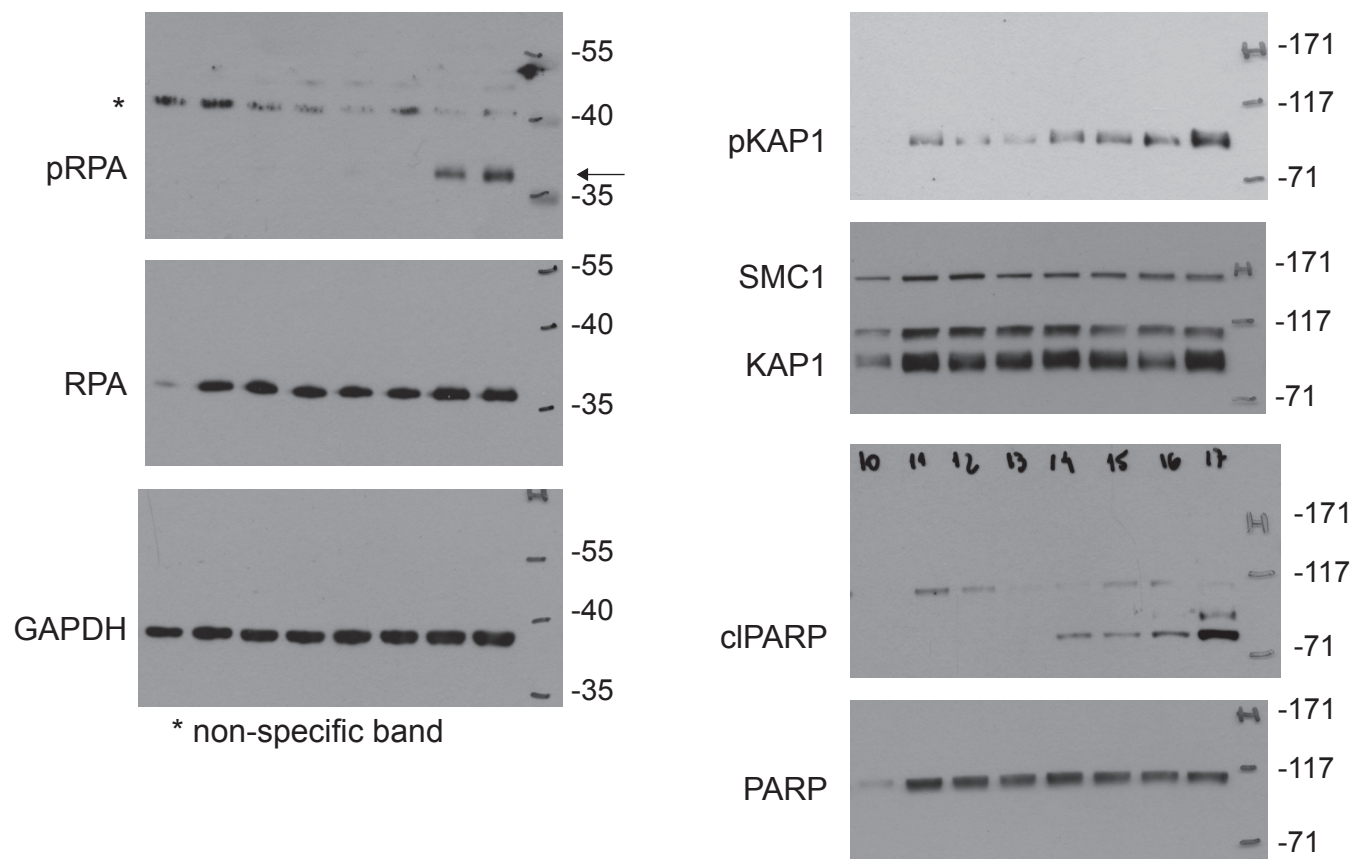

B

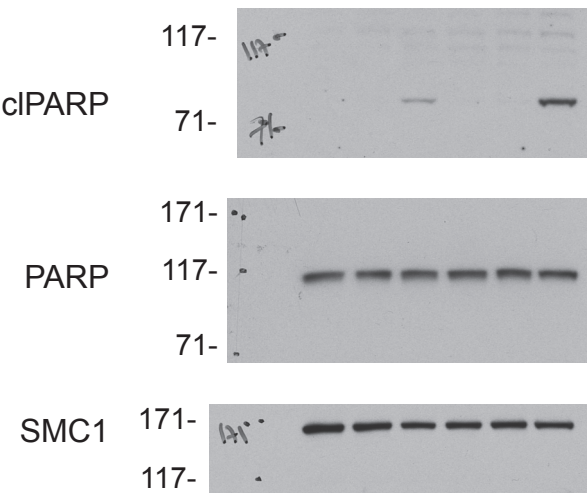

Supplement: Supplementary file 3 — Source Data for Appendix [file EMMM-11-e9982-s005.zip › appenidx_source_data/Appendix_Fig_S3_source_data.pdf]

Source data - Appendix Figure S4

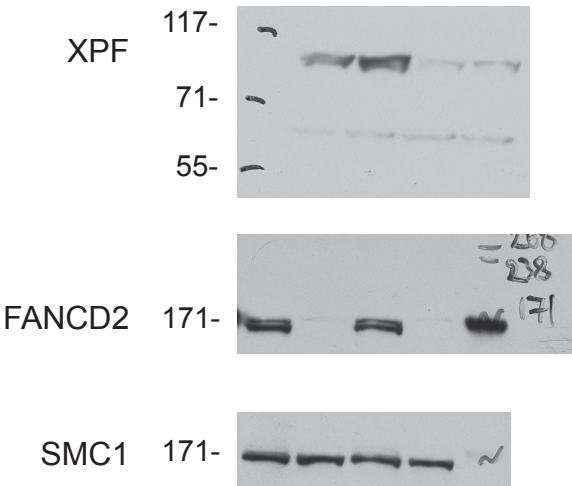

Supplement: Supplementary file 3 — Source Data for Appendix [file EMMM-11-e9982-s005.zip › appenidx_source_data/Appendix_Fig_S4_source_data.pdf]

Source data - Figure 3

**A**

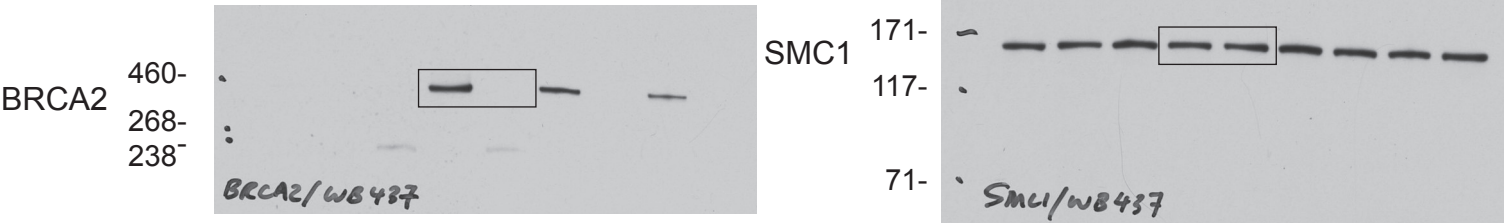

**B**

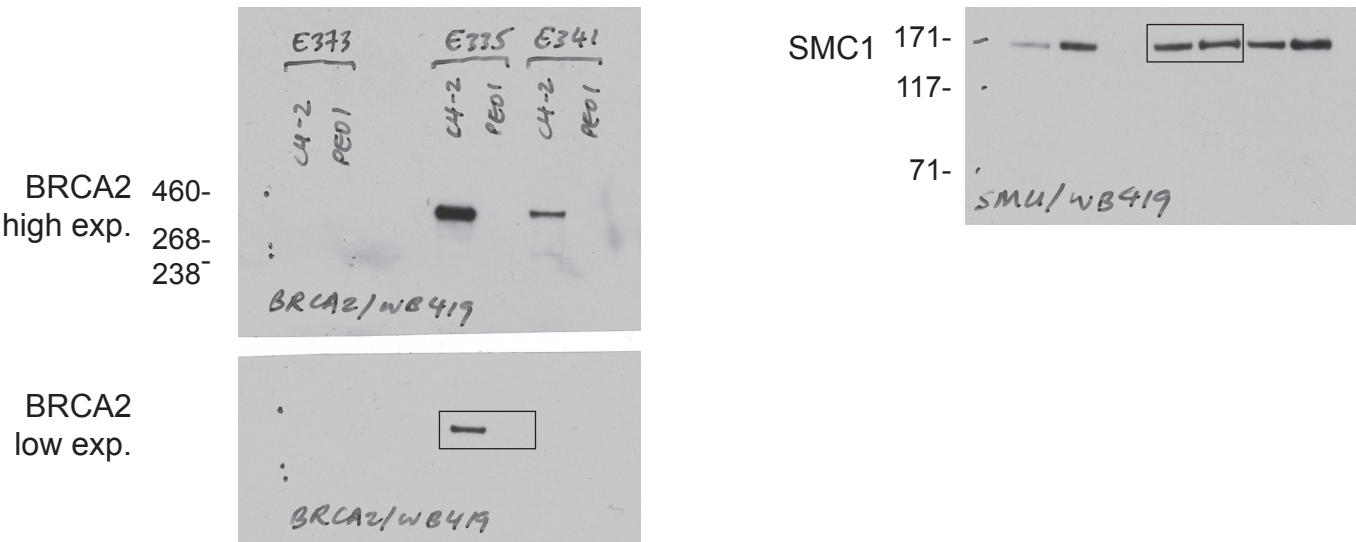

**C**

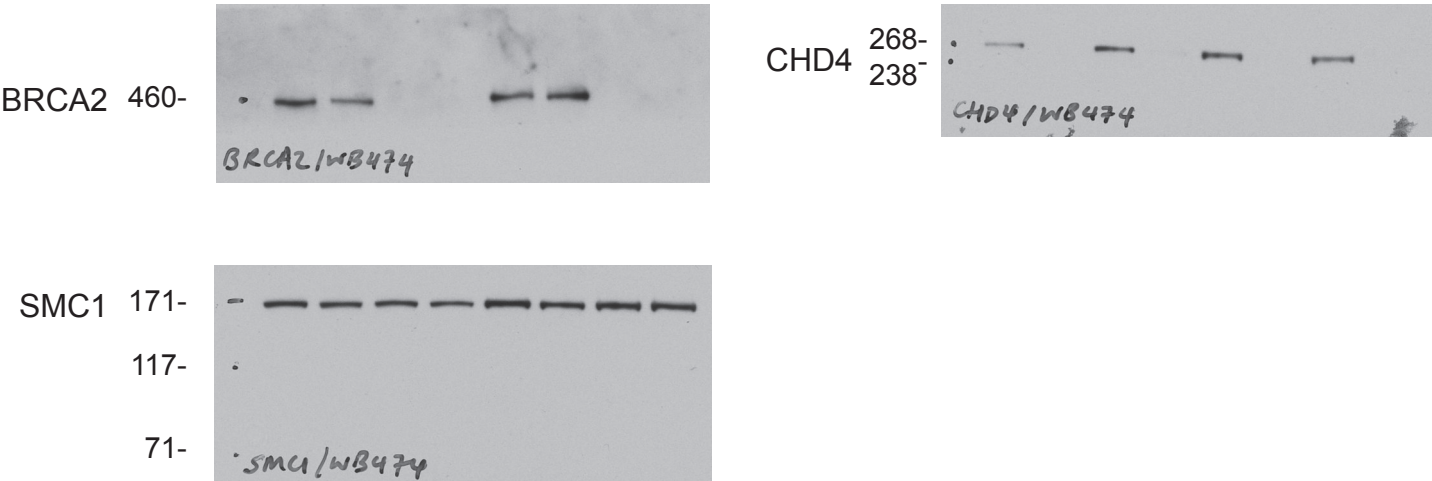

Supplement: Supplementary file 5 — Source Data for Figure 3 [file EMMM-11-e9982-s003.pdf]

Source data - Figure 4

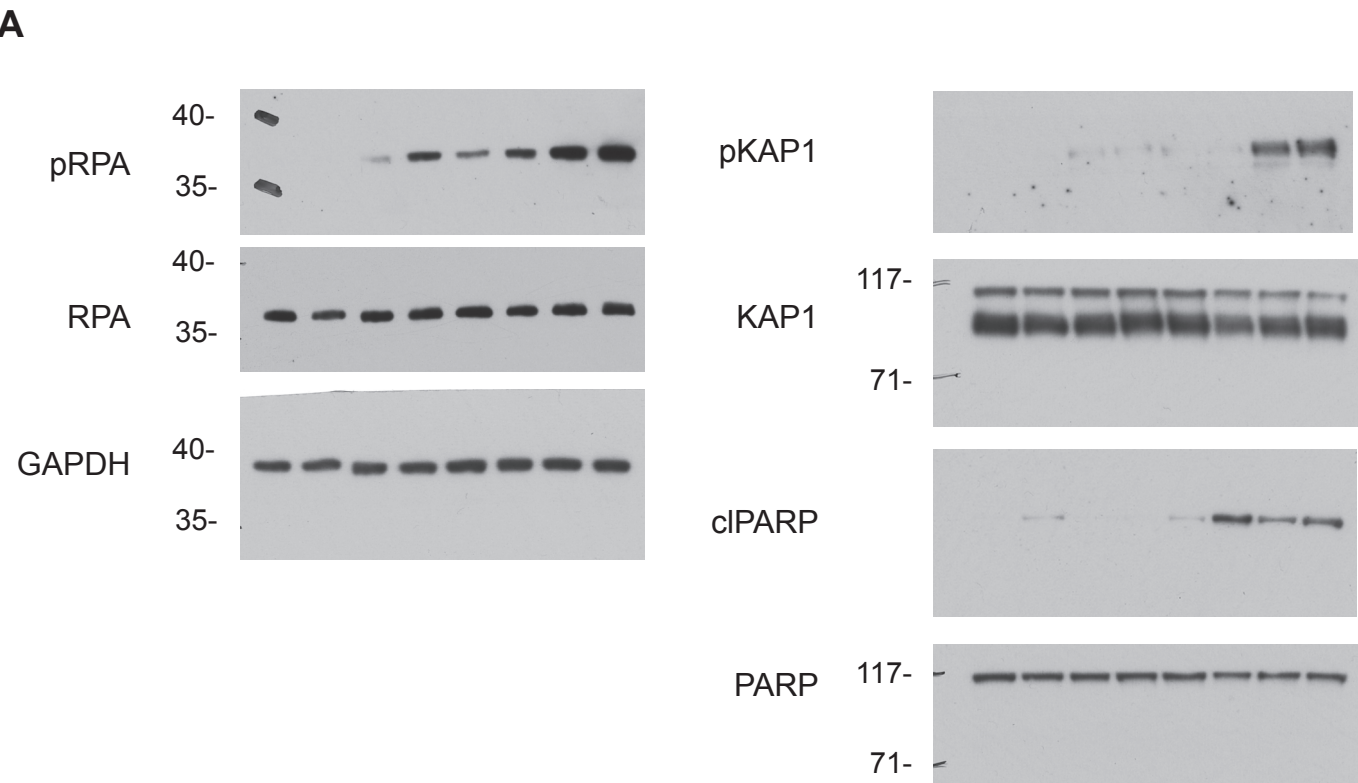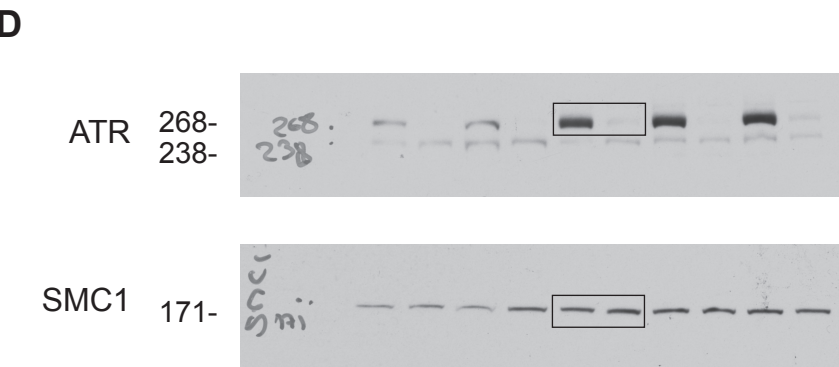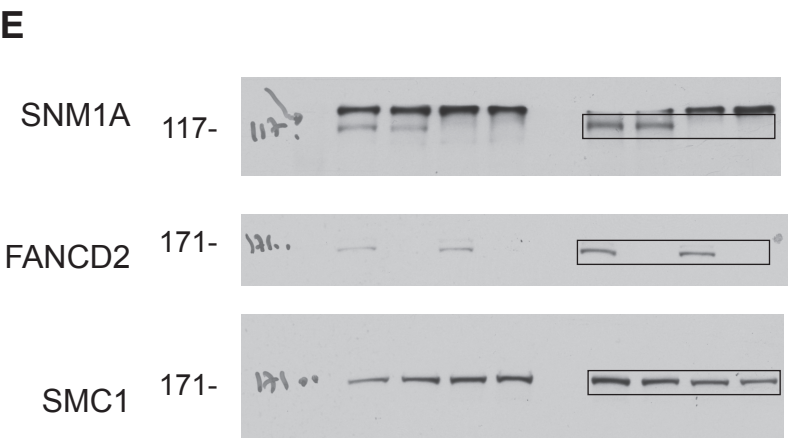

Supplement: Supplementary file 6 — Source Data for Figure 4 [file EMMM-11-e9982-s004.pdf]
